# Atypical Social Development in Vasopressin-Deficient Brattleboro Rats^1^,^2^,^3^

**DOI:** 10.1523/ENEURO.0150-15.2016

**Published:** 2016-04-06

**Authors:** Matthew J. Paul, Nicole V. Peters, Mary K. Holder, Anastasia M. Kim, Jack Whylings, Joseph I. Terranova, Geert J. de Vries

**Affiliations:** 1 Psychology Department, University at Buffalo, SUNY, Buffalo, New York 14260; 2 Neuroscience Department, Georgia State University, Atlanta, Georgia 30302

**Keywords:** play behavior, postnatal development, social behavior, ultrasonic vocalizations, vasopressin

## Abstract

Over the past 3 decades, a large body of evidence has accumulated demonstrating that the neuropeptide arginine vasopressin (AVP) plays a critical role in regulating social behavior. The overwhelming majority of this evidence comes from adults, leaving a gap in our understanding of the role of AVP during development. Here, we investigated the effect of chronic AVP deficiency on a suite of juvenile social behaviors using Brattleboro rats, which lack AVP due to a mutation in the *Avp* gene. Social play behavior, huddling, social investigation & allogrooming, and ultrasonic vocalizations (USVs) of male and female rats homozygous for the Brattleboro mutation (Hom) were compared with their wild-type (WT) and heterozygous (Het) littermates during same-sex, same-genotype social interactions. Male and female Hom juveniles exhibited less social play than their Het and WT littermates throughout the rise, peak, and decline of the developmental profile of play. Hom juveniles also emitted fewer prosocial 50 kHz USVs, and spectrotemporal characteristics (call frequency and call duration) of individual call types differed from those of WT and Het juveniles. However, huddling behavior was increased in Hom juveniles, and social investigation and 22 kHz USVs did not differ across genotypes, demonstrating that not all social interactions were affected in the same manner. Collectively, these data suggest that the *Avp* gene plays a critical role in juvenile social development.

## Significance Statement

Several neurodevelopmental disorders are characterized by deficits in social behaviors, the underlying neurobiology of which is not understood. Arginine vasopressin (AVP) has emerged as a candidate neuropeptide through which two such groups of disorders, autism spectrum disorders and schizophrenia, might impact social function. Nonetheless, only a few studies have investigated the role of AVP in social development. Here, we find that rats with a mutation in the *Avp* gene exhibit “atypical” juvenile social behaviors and vocal communication. These findings suggest that AVP plays a critical role in the regulation of the quantity, quality, and type of social behaviors expressed during development.

## Introduction

Childhood and adolescence are periods of marked social development, when individuals acquire the necessary skills for independence (for review, see Spear, 2000). The most prominent social behavior of juveniles across many species is social play, where individuals engage in mock fighting behavior (Bekoff and Byers, 1998; Pellis and Pellis, 1998). In rats, social play emerges during the juvenile phase (∼18 d of age), peaks during early adolescence (∼35 d of age), and declines thereafter (Panksepp, 1981; Pellis and Pellis, 1990). This well characterized developmental profile makes play ideal for studying juvenile and adolescent social development. Furthermore, play contributes to social and emotional development (Pellegrini, 1988; Vanderschuren et al., 1997; Hol et al., 1999; van den Berg et al., 1999). During social interactions, such as play, rats emit ultrasonic vocalizations (USVs) as a form of affective communication (for review, see Wöhr and Schwarting, 2013). Calls with frequencies close to 50 kHz are thought to signal positive affect, whereas ∼22 kHz calls are thought to signal distress (for review, see Brudzynski, 2013). Infant rats and mice also emit ∼40 kHz calls when separated from their mother (for review, see Scattoni et al., 2009).

Many neurodevelopmental disorders are characterized by deficits in social behaviors such as play and communication (e.g., autism spectrum disorders, schizophrenia, and attention deficit hyperactivity disorder; Alessandri, 1992; Jones et al., 1994; Jordan, 2003; Scattoni et al., 2009). Uncovering the underlying neurobiology by which neurodevelopmental disorders impact social function is a difficult task, especially given that the neural mechanisms that regulate “normal” social development are not understood. Here, we focus on the role of arginine vasopressin (AVP) in social development. This peptide is often referred to as a “social neuropeptide” because of its actions on a number of social and antisocial behaviors, including pair bonding, parental behaviors, social recognition, flank marking, and aggression (for review, see Caldwell et al., 2008; Albers, 2012; Bosch and Neumann, 2012). The overwhelming majority of this research has been conducted in adults, but emerging evidence indicates that AVP also influences juvenile social behaviors. The most direct evidence comes from intracranial injections of AVP agonists or antagonists, which impact social play (Cheng and Delville, 2009; Veenema et al., 2013), social recognition (Veenema et al., 2012), and USVs (Lukas and Wöhr, 2015) of juvenile rodents. While these findings provide strong evidence that AVP influences the immediate expression of juvenile social behaviors, the direction of the effects often depends on the age and sex of the subjects, the context of the experiment, and the brain area injected (Veenema et al., 2012, 2013; Bredewold et al., 2014). Hence, we do not yet understand the role of AVP in social development.

Brattleboro rats provide a unique model to study the effects of lifelong deficits in AVP on social behaviors. These rats have a single base pair deletion in exon 2 of the *Avp* gene that disrupts the production of AVP (Schmale and Richter, 1984). The behavior of adult homozygous Brattleboro (Hom) rats has been well studied, and deficits have been found in the major functions assigned to AVP, including social behaviors such as social recognition/discrimination (Engelmann and Landgraf, 1994; Feifel et al., 2009) and social interactions (Lin et al., 2013). Studies on the behavioral development of Brattleboro rats have been confined to early postnatal life (first 2 weeks of life; Zelena et al., 2008; Lin et al., 2013). Infant Hom rats exhibit decreased aggregation (Schank, 2009) and emit fewer maternal separation-induced USVs (Varga et al., 2015), suggesting that the development of social behaviors might be affected by the Brattleboro mutation. Juvenile social development has not been studied in Brattleboro rats.

In the present study, we test the impact of chronic AVP deficiency on juvenile social development by assessing the effects of the Brattleboro mutation on several social behaviors (social play, USVs, huddling, and social investigation & allogrooming). We find that male and female AVP-deficient Hom rats exhibit lower levels of social play at all stages of play development (onset, peak, and decline of play). Juvenile Hom rats also emit fewer 50 kHz USVs with altered spectrotemporal characteristics. Not all social behaviors are affected in the same manner, however, as juvenile Hom rats display more huddling episodes, and social investigation & allogrooming do not differ between genotypes. These data demonstrate that deficits in AVP throughout development impact the quantity and quality of juvenile social interactions and communication.

## Materials and Methods

### Animals and housing conditions

A colony of Brattleboro rats (with Long–Evans background) was established in our laboratory from rats purchased from the Rat Resource and Research Center (University of Missouri, Columbia, MO). Brattleboro rats were housed in either opaque plastic cages with Carefresh bedding and wood chips (48 × 27 × 20 cm) or ventilated transparent OptiRat plastic cages with Bed-O-Cobs bedding (35.6 × 48.5 × 21.8 cm). For all experiments, the day of birth was considered postnatal day 0 (P0). Room lights were set to a 12 h light/12 h dark cycle (lights off at 5:00 P.M. Eastern time), and ambient temperature was maintained at 23°C. Food and water were available *ad libitum*. All procedures were performed in accordance with the *Guide for the Care and Use of Laboratory Animals* and were approved by the Animal Care and Use Committee at Georgia State University and the University of Massachusetts, Amherst.

### Experiment 1: emergence of play behavior in Brattleboro rats

Wild-type (WT), heterozygous (Het), and homozygous (Hom) Brattleboro offspring were obtained from Het × Het breeding pairs from our colony. Overall, the distribution of genotypes was 1.3/2.0/0.9 (WT/Het/Hom). Each rat pup was tested for play behavior once at P17, P19, P21, or P23. All rats were tested prior to weaning, which occurred at P24. Rats were removed from their litters 2–3 h before being paired with a similarly treated age-matched, same-genotype, same-sex rat in a clean cage for a social behavior test (Panksepp, 1981; Paul et al., 2014).

### Experiment 2: social behaviors and ultrasonic vocalizations of juvenile Brattleboro rats

WT, Het, and Hom Brattleboro offspring were obtained from Het × Het breeding pairs from our colony. Overall, the distribution of genotypes was 0.9/2.0/0.9 (WT/Het/Hom). Rats were weaned at P22, at which point they were housed with an age-matched, same-genotype, same-sex cage mate. At P33 (±2 d) or P43 (±2 d), cage mates were single housed for ∼24 h before being reunited in a social behavior test the following day.

### Social behavior tests

All tests were conducted within the first 2.5 h of lights off under red light. Animals were paired with an age-matched, same-sex, same-genotype playmate in a fresh cage for 20 min, and their behavior was videotaped. In Experiment 2, bedding was removed from the test cage to minimize background noise interference with the ultrasonic recordings. Play attacks (i.e., lunges toward the nape of the playmate’s neck), pins (i.e., animal lying in the supine position with playmate on top), and boxing events (i.e., both animals standing on their hindpaws and pushing each other with their forepaws), as described in studies by Meaney and Stewart (1981a) and Vanderschuren et al. (1997), were scored by a researcher who was blind to the treatment conditions using JWatcher software (http://www.jwatcher.ucla.edu/; Experiment 1) or The Observer XT11 (Noldus Information Technology Inc.; Experiment 2). In Experiment 2, the number of social investigation & allogrooming events and huddling episodes were also scored using The Observer XT11.

### Ultrasonic vocalization recordings

Vocal emissions were recorded for the duration of the social behavior tests using an UltraSoundGate CM16/CMPA microphone (Avisoft Bioacoustics) placed just above the testing cage. The microphone was connected to a computer via an Avisoft Bioacoustics UltraSoundGate 116Hb. Acoustic data were recorded with a sampling rate of 250 kHz in 16 bit format, and spectrograms were constructed by fast Fourier transformation (FFT; 256 FFT length, 100% frame, FlatTop window, and 50% time window overlap; SASLab Pro, Avisoft Bioacoustics). All USVs made within the first 10 min of the play behavior trial were manually marked by investigators who were blind to the age, sex, and genotype of the rats. In order to be marked, calls had to be at least 10 ms in length, and distinct calls had to be separated by at least 10 ms. Several call parameters were quantified, including fundamental frequency, duration, and number of calls emitted. Call frequency (in hertz) was calculated by averaging the fundamental frequency at call onset, call offset, and peak amplitude of the call (integrated frequency). A subset (20% random sampling) of the calls was selected and manually classified into the 15 call categories described in the study by Wright et al. (2010).

### Genotyping of Brattleboro rats

The Brattleboro mutation is a single base pair deletion in exon 2 of the *Avp* gene that disrupts processing of the AVP prohormone (Schmale and Richter, 1984). Previous genotyping protocols required DNA sequencing after PCR amplification to detect the single base pair deletion. In the present study, we developed a faster and cheaper method, replacing the sequencing step with restriction enzyme digestion followed by gel electrophoresis. Tail tissue was harvested from rat pups between 8 and 12 d of age using ice-cold ethanol as a local anesthetic. For animals in Experiment 1, tails were digested at 55°C overnight in 400 μl of Tail Lysis Buffer containing 4 μl of Proteinase K. DNA was extracted and purified with phenol, chloroform:isoamyl alcohol (24:1), isopropanol, and 70% ethanol. For animals in Experiment 2, the REDExtract-N-Amp Tissue PCR Kit (Sigma-Aldrich) was used for tail digestion and DNA extraction. DNA surrounding the base pair deletion was amplified by PCR using the following primers: forward, GACGAGCTGGGCTGCTTC; reverse, CCTCAGTCCCCCACTTAGCC. Twenty microliters of PCR product was then digested with BcgI restriction endonuclease (New England BioLabs) at 37°C overnight using the following concentrations: 3 μl of 10× NEBuffer 3, 4 μl of 10× S-adenosylmethionine, 2 μl of nuclease-free water, and 1 μl of BcgI. BcgI recognizes and cuts only the mutant Brattleboro PCR product, resulting in two DNA fragments of similar size (92 and 97 bp). Therefore, samples from WT rats exhibit a single 222 bp band after gel electrophoresis, whereas those of Hom rats exhibit a single ∼95 bp band (the two fragments do not separate on a 2% agarose gel). Samples from Het rats exhibit both WT and Hom bands. To validate this procedure, we confirmed the genotyping results from a subset of samples with the traditional sequencing methodology.

### Statistical analyses

Data were analyzed by three-way ANOVA with genotype, age, and sex as the independent variables. Because the main effect of age was significant for the overwhelming majority of measures (22 of 27), data were also analyzed separately at each age by two-way ANOVA, and this analysis was depicted in all figures. Where the main effect of genotype was significant, and in a few cases where it approached significance (*p* = 0.06), *post hoc* comparisons between each genotype were assessed using Fisher’s PLSD. For a number of comparisons, the data were not normally distributed due to the high number of zero values, and the distribution could not be corrected by using a log(*x* + 0.01) transformation (Tables 1–3 located at the end of the article). This is to be expected when age groups prior to the onset of the behavior are included in the analysis (in Experiment 1) and when one assesses behaviors not highly expressed during motivated social behavior tests (e.g., distress USVs and huddling in Experiment 2). Although multifactorial ANOVA is robust when the data are not normally distributed, we confirmed significant main effects and *post hoc* comparisons from non-normally distributed data with the appropriate nonparametric test (Kruskal–Wallis or Mann–Whitney *U* tests). For all of these comparisons, except for *post hoc* tests assessing age differences in Experiment 1, the ANOVA and nonparametric tests yielded the same result. For the *post hoc* tests where the results differed, we reported the nonparametric statistic. Significance was assumed at *p* < 0.05.

**Table 1:** Experiment 1 statistical analyses

	Data structure	Dependent variable	Comparison	Type of test	*p* value	Power
a1	Non-normal distribution	Total play	Main effect of age	Three-way ANOVA, K-W	<0.0001 (ANOVA and K-W)	1.000
a2	Non-normal distribution		Main effect of genotype	Three-way ANOVA, K-W	0.0310 (ANOVA), 0.0375 (K-W)	0.649
a3	Non-normal distribution		Main effect of sex	Three-way ANOVA, M-W	0.9342 (ANOVA), 0.3852 (M-W)	0.051
a4	Non-normal distribution		P17 vs P19	M-W	<0.0001	1.000
a5	Non-normal distribution		P19 vs P21	M-W	<0.0001	1.000
a6	Non-normal distribution		Hom vs WT	Fisher's PLSD, M-W	0.0024 (Fisher's), 0.0124 (M-W)	0.804
a7	Non-normal distribution		Hom vs Het	Fisher's PLSD, M-W	0.0079 (Fisher's), 0.043 (M-W)	0.612
a8	Normal distribution		Main effect of genotype, P21	Two-way ANOVA	0.0021	0.918
a9	Normal distribution		Hom vs WT, P21	Fisher's PLSD	0.0004	0.934
a10	Normal distribution		Hom vs Het, P21	Fisher's PLSD	0.0604	0.610
a11	Normal distribution		Het vs WT, P21	Fisher's PLSD	0.0342	0.470
a12	Normal distribution		Main effect of genotype, P23	Two-way ANOVA	0.0582	0.550
a13	Normal distribution		Hom vs WT, P23	Fisher's PLSD	0.0876	0.526
a14	Normal distribution		Hom vs Het, P23	Fisher's PLSD	0.0190	0.604
a15	Normal distribution		Main effect of genotype, P19	Two-way ANOVA	0.6717 (ANOVA)	0.110
b1	Non-normal distribution	Pins	Main effect of age	Three-way ANOVA, K-W	<0.0001 (ANOVA and K-W)	1.000
b2	Non-normal distribution		Main effect of genotype	Three-way ANOVA, K-W	0.0118 (ANOVA), 0.0046 (K-W)	0.773
b3	Non-normal distribution		Main effect of sex	Three-way ANOVA, M-W	0.9655 (ANOVA), 0.5681 (M-W)	0.050
b4	Non-normal distribution		P17 vs P19	M-W	<0.0001	1.000
b5	Non-normal distribution		P19 vs P21	M-W	<0.0001	1.000
b6	Non-normal distribution		Hom vs WT	Fisher's PLSD, M-W	0.0003 (Fisher's), 0.0015 (M-W)	0.950
b7	Non-normal distribution		Hom vs Het	Fisher's PLSD, M-W	0.0019 (Fisher's), 0.0085 (M-W)	0.797
b8	Normal distribution		Main effect of genotype, P21	Two-way ANOVA	0.0015	0.933
b9	Normal distribution		Hom vs WT, P21	Fisher's PLSD	0.0003	0.942
b10	Normal distribution		Hom vs Het, P21	Fisher's PLSD	0.0436	0.764
b11	Normal distribution		Het vs WT, P21	Fisher's PLSD	0.0363	0.436
b12	Normal distribution		Main effect of genotype, P23	Two-way ANOVA	0.0280	0.667
b13	Normal distribution		Hom vs WT, P23	Fisher's PLSD	0.0377	0.770
b14	Normal distribution		Hom vs Het, P23	Fisher's PLSD	0.0099	0.691
b15	Non-normal distribution		Main effect of genotype, P19	Two-way ANOVA, K-W	0.3325 (ANOVA), 0.3213 (K-W)	0.230
c1	Non-normal distribution	Play attacks	Main effect of age	Three-way ANOVA, K-W	<0.0001 (ANOVA and K-W)	1.000
c2	Non-normal distribution		Main effect of genotype	Three-way ANOVA, K-W	0.0629 (ANOVA), 0.0887 (K-W)	0.537
c3	Non-normal distribution		Main effect of sex	Three-way ANOVA, M-W	0.9314 (ANOVA), 0.4056 (M-W)	0.051
c4	Non-normal distribution		P17 vs P19	M-W	<0.0001	1.000
c5	Non-normal distribution		P19 vs P21	M-W	<0.0001	1.000
c6	Non-normal distribution		Hom vs WT	Fisher's PLSD, M-W	0.0086 (Fisher's), 0.0318 (M-W)	0.645

K-W, Kruskal–Wallis test; M-W, Mann–Whitney *U* test.

**Table 2:** Experiment 2 statistical analyses

	Data structure	Dependent variable	Comparison	Type of test	*p* value	Power
d1	Normal distribution	Total play	Main effect of age	Three-way ANOVA	<0.0001	0.997
d2	Normal distribution		Main effect of genotype	Three-way ANOVA	<0.0001	1.000
d3	Normal distribution		Main effect of sex	Three-way ANOVA	0.6259	0.076
d4	Normal distribution		Hom vs WT	Fisher's PLSD	<0.0001	1.000
d5	Normal distribution		Hom vs Het	Fisher's PLSD	<0.0001	1.000
e1	Non-normal distribution	Pins	Main effect of age	Three-way ANOVA, M-W	0.0041 (ANOVA), 0.0002 (M-W)	0.843
e2	Non-normal distribution		Main effect of genotype	Three-way ANOVA, K-W	0.0031 (ANOVA), <0.0001 (K-W)	0.890
e3	Non-normal distribution		Main effect of sex	Three-way ANOVA, M-W	0.9169 (ANOVA), 0.3571 (M-W)	0.051
e4	Non-normal distribution		Hom vs WT	Fisher's PLSD, M-W	0.0048 (Fisher's), <0.0001 (M-W)	0.908
e5	Non-normal distribution		Hom vs Het	Fisher's PLSD, M-W	0.0016 (Fisher's), <0.0001 (M-W)	0.903
e6	Non-normal distribution		genotype x sex, P44	Two-way ANOVA	0.0076	0.826
e7	Normal distribution		Het male vs Het female, P44	Fisher's PLSD	0.0019	0.921
f1	Normal distribution	Play attacks	Main effect of age	Three-way ANOVA	<0.0001	0.999
f2	Normal distribution		Main effect of genotype	Three-way ANOVA	<0.0001	1.000
f3	Normal distribution		Main effect of sex	Three-way ANOVA	0.5730	0.085
f4	Normal distribution		Hom vs WT	Fisher's PLSD	<0.0001	1.000
f5	Normal distribution		Hom vs Het	Fisher's PLSD	<0.0001	1.000
g1	Normal distribution	Total social behaviors	Main effect of age	Three-way ANOVA	<0.0001	1.000
g2	Normal distribution		Main effect of genotype	Three-way ANOVA	<0.0001	1.000
g3	Normal distribution		Main effect of sex	Three-way ANOVA	0.8892	0.052
g4	Normal distribution		Hom vs WT	Fisher's PLSD	<0.0001	1.000
g5	Normal distribution		Hom vs Het	Fisher's PLSD	<0.0001	1.000
h1	Normal distribution	Social Investigation/allogrooming	Main effect of age	Three-way ANOVA	0.0090	0.758
h2	Normal distribution		Main effect of genotype	Three-way ANOVA	0.5137	0.156
h3	Normal distribution		Main effect of sex	Three-way ANOVA	0.4631	0.109
i1	Non-normal distribution	Huddling	Main effect of age	Three-way ANOVA, M-W	<0.0001 (ANOVA), 0.0001 (M-W)	0.999
i2	Non-normal distribution		Main effect of genotype	Three-way ANOVA, K-W	<0.0001 (ANOVA), <0.0001 (K-W)	1.000
i3	Non-normal distribution		Main effect of sex	Three-way ANOVA, M-W	0.8084 (ANOVA), 0.8321 (M-W)	0.057
i4	Normal distribution		Hom vs WT	Fisher's PLSD, M-W	<0.0001 (Fisher's), 0.0003 (M-W)	0.948
i5	Non-normal distribution		Hom vs Het	Fisher's PLSD, M-W	<0.0001 (Fisher's), <0.0001 (M-W)	1.000
j1	Normal distribution	All USVs	Main effect of genotype	Three-way ANOVA	<0.0001	0.993
j2	Normal distribution		Hom vs WT	Fisher's PLSD	0.0020	0.942
j3	Normal distribution		Hom vs Het	Fisher's PLSD	<0.0001	0.998
k1	Normal distribution	50 kHz USVs	Main effect of age	Three-way ANOVA	0.0380	0.537
k2	Normal distribution		Main effect of genotype	Three-way ANOVA	0.0001	0.990
k3	Normal distribution		Hom vs WT	Fisher's PLSD	0.0022	0.947
k4	Normal distribution		Hom vs Het	Fisher's PLSD	<0.0001	0.996
l1	Non-normal distribution	22 kHz USVs	Main effect of age	Three-way ANOVA, M-W	0.0001 (ANOVA), <0.0001 (M-W)	0.986
l2	Non-normal distribution		Main effect of genotype	Three-way ANOVA, K-W	0.2541 (ANOVA), 0.2262 (K-W)	0.282

K-W, Kruskal–Wallis test; M-W, Mann–Whitney *U* test.

**Table 3:** USV call type statistical analyses

	Data structure	Dependent variable	Comparison	Type of test	Exact *p* value	Power
m1	Normal distribution	Complex calls	Main effect of age	Three-way ANOVA	0.1349	0.304
m2	Normal distribution	(Number)	Main effect of genotype	Three-way ANOVA	0.0258	0.676
m3	Normal distribution		Hom vs WT	Fisher's PLSD	0.0303	0.594
m4	Normal distribution		Hom vs Het	Fisher's PLSD	0.0116	0.796
m5	Normal distribution		Main effect of genotype, P34	Two-way ANOVA	0.1603	0.366
m6	Normal distribution		Main effect of genotype, P44	Two-way ANOVA	0.1587	0.367
n1	Normal distribution	Upward-ramp calls	Main effect of age	Three-way ANOVA	0.0028	0.875
n2	Normal distribution	(Number)	Main effect of genotype, P34	Two-way ANOVA	0.0185	0.725
n3	Normal distribution		Hom vs WT, P34	Fisher's PLSD	0.0112	0.724
n4	Normal distribution		Hom vs Het, P34	Fisher's PLSD	0.0163	0.755
n5	Normal distribution		Main effect of genotype, P44	Two-way ANOVA	0.6322	0.120
o1	Normal distribution	Flat calls	Main effect of age	Three-way ANOVA	0.8419	0.054
o2	Normal distribution	(Number)	Main effect of genotype, P34	Two-way ANOVA	0.0270	0.673
o3	Normal distribution		Hom vs WT, P34	Fisher's PLSD	0.0574	0.520
o4	Normal distribution		Hom vs Het, P34	Fisher's PLSD	0.0056	0.832
o5	Normal distribution		Main effect of genotype, P44	Two-way ANOVA	0.5538	0.141
p1	Normal distribution	Step-up calls	Main effect of age	Three-way ANOVA	0.0355	0.549
p2	Normal distribution	(Number)	Main effect of genotype, P34	Two-way ANOVA	0.0058	0.850
p3	Normal distribution		Hom vs WT, P34	Fisher's PLSD	0.0084	0.857
p4	Normal distribution		Hom vs Het, P34	Fisher's PLSD	0.0022	0.918
p5	Normal distribution		Main effect of genotype, P44	Two-way ANOVA	0.0543	0.562
q1	Non-normal distribution	Trills	Main effect of age	Three-way ANOVA, M-W	0.0144 (ANOVA), 0.0025 (M-W)	0.694
q2	Non-normal distribution	(Number)	Main effect of sex	Three-way ANOVA, K-W	0.0046 (ANOVA), <0.0001 (K-W)	0.830
q3	Normal distribution		Main effect of genotype, P34	Two-way ANOVA	0.0092	0.807
q4	Normal distribution		Hom vs WT, P34	Fisher's PLSD	0.0978	0.789
q5	Normal distribution		Hom vs Het, P34	Fisher's PLSD	0.0045	0.746
q6	Normal distribution		Main effect of genotype, P44	Two-way ANOVA	0.0009	0.954
q7	Normal distribution		Hom vs WT, P44	Fisher's PLSD	0.0300	0.887
q8	Normal distribution		Hom vs Het, P44	Fisher's PLSD	0.0005	0.923
r1	Normal distribution	Complex calls	Main effect of genotype	Three-way ANOVA	0.0142	0.754
r2	Normal distribution	(Int. Freq.)	Hom vs WT	Fisher's PLSD	0.1043	0.414
r3	Normal distribution		Hom vs Het	Fisher's PLSD	0.0053	0.703
r4	Normal distribution		Main effect of age	Three-way ANOVA	<0.0001	0.999
r5	Normal distribution		age x sex	Three-way ANOVA	0.9431	0.059
s1	Normal distribution	Upward-ramp calls	Main effect of genotype	Three-way ANOVA	0.0002	0.986
s2	Normal distribution	(Int. Freq.)	Hom vs WT	Fisher's PLSD	0.0007	0.936
s3	Normal distribution		Hom vs Het	Fisher's PLSD	<0.0001	0.990
s4	Normal distribution		Main effect of age	Three-way ANOVA	<0.0001	0.994
s5	Normal distribution		age x sex	Three-way ANOVA	0.6940	0.106
t1	Normal distribution	Flat calls	Main effect of genotype	Three-way ANOVA	0.0020	0.916
t2	Normal distribution	(Int. Freq.)	Hom vs WT	Fisher's PLSD	0.0022	0.930
t3	Normal distribution		Hom vs Het	Fisher's PLSD	0.0014	0.825
t4	Normal distribution		Main effect of age	Three-way ANOVA	<0.0001	0.999
t5	Normal distribution		age x sex	Three-way ANOVA	0.8255	0.079
u1	Normal distribution	Step-up calls	Main effect of genotype	Three-way ANOVA	0.0186	0.721
u2	Normal distribution	(Int. Freq.)	Hom vs WT	Fisher's PLSD	0.0416	0.531
u3	Normal distribution		Hom vs Het	Fisher's PLSD	0.0066	0.706
u4	Normal distribution		Main effect of age	Three-way ANOVA	0.0416	0.521
u5	Normal distribution		age x sex	Three-way ANOVA	0.0588	0.548
v1	Normal distribution	Trills	Main effect of genotype	Three-way ANOVA	0.2452	0.288
v2	Normal distribution	(Int. Freq.)	Main effect of age	Three-way ANOVA	0.1801	0.252
w1	Normal distribution	Step-up calls	Main effect of genotype, P44	Two-way ANOVA	0.0026	0.908
w2	Normal distribution	(Dur.)	Hom vs WT, P44	Fisher's PLSD	0.0195	0.592
w3	Normal distribution		Hom vs Het, P44	Fisher's PLSD	0.0006	0.947
w4	Normal distribution		Main effect of age	Three-way ANOVA	0.2480	0.198
x1	Normal distribution	Trills	Main effect of genotype, P44	Two-way ANOVA	0.0090	0.810
x2	Normal distribution	(Dur.)	Hom vs WT, P44	Fisher's PLSD	0.0040	0.799
x3	Normal distribution		Het vs WT, P44	Fisher's PLSD	0.0157	0.659
x4	Normal distribution		Hom vs Het, P44	Fisher's PLSD	0.2632	0.213
x5	Normal distribution		Main effect of age	Three-way ANOVA	0.1010	0.357
y1	Normal distribution	Complex calls (Dur.)	Main effect of age	Three-way ANOVA	0.0210	0.638
z1	Normal distribution	Upward-ramp calls (Dur.)	Main effect of age	Three-way ANOVA	0.0078	0.775
aa1	Normal distribution	Flat calls (Dur.)	Main effect of age	Three-way ANOVA	0.0036	0.854

K-W, Kruskal–Wallis test; M-W, Mann–Whitney *U* test.

## Results

### Experiment 1: emergence of play behavior in Brattleboro rats

**Figure 1.**
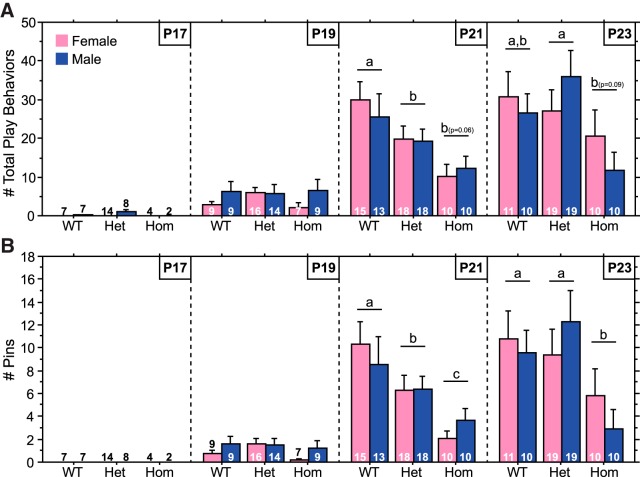
Hom Brattleboro weanlings play less than their WT and Het littermates. ***A***, ***B***, Number of total play behaviors (***A***) and pins (***B***) of Hom, Het, and WT rats during a 20 min test at P17, P19, P21, or P23. Sample sizes are indicated within each bar. Data from each age were obtained from separate cohorts of animals. Genotypes with differing letters differ significantly from each other (*p* < 0.05, Fisher’s PLSD); where differences approach significance, the *p* value is included in parentheses next to the letter representing the appropriate comparison. See Results for ANOVA details.

Consistent with other reports (Panksepp, 1981; Paul et al., 2014), the developmental onset of play occurred at ∼P19–P21 (Fig. 1). Play behavior was virtually absent at P17, increased slightly at P19, and further increased at P21 (main effect of age, *p* < 0.0001 for total play^a1^, pins^b1^, and pounces^c1^; P17 vs P19 and P19 vs P21, *p* < 0.0001, for total play^a4,a5^, pins^b4,b5^, and play attacks^c4,c5^; superscripts used here and further in the document indicate rows in Tables 1–3 located at the end of the article). Play behavior of males and females did not differ (main effect of sex, *p* > 0.38 for total play^a3^, pins^b3^, and play attacks^c3^).

Overall, Hom rats played less than their WT and Het littermates (Fig. 1*A*; total play, main effect of genotype, *p* < 0.04^a2^; Hom vs WT^a6^ or Het^a7^, *p* < 0.05). This was due to fewer numbers of pins (Fig. 1*B*; main effect of genotype, *p* < 0.02^b2^; Hom vs WT^b6^ or Het^b7^, *p* < 0.009) and play attacks: while the main effect of genotype fell short of significance for play attacks (*p* = 0.06 for ANOVA and *p* = 0.09 for Kruskal–Wallis test^c2^), *post hoc* comparisons indicated that Hom weanlings did exhibit fewer play attacks than WT weanlings (*p* < 0.04^c6^; not illustrated). Boxing events were rare in all genotypes, with <0.25 events during the 20 min test for all groups; no Hom pairs exhibited a boxing event. Decreased total play and pins of Hom rats were evident at P21 (main effect of genotype, *p* < 0.003 for both behaviors^a8,b8^; Hom vs WT, *p* < 0.0005 for both behaviors^a9,b9^) and P23, although comparisons for total play fell short of significance at P23 (main effect of genotype, *p* < 0.03 for pins^b12^, *p* = 0.06 for total play^a12^; Hom vs WT, *p* < 0.04 for pins^b13^, *p* = 0.09 for total play^a13^). Notably, Het animals exhibited less total play and fewer pins than WT animals at P21 (Het vs WT, *p* < 0.04 for both behaviors^a11,b11^, Fisher’s PLSD). Genotype did not impact total play or pins at P19 (main effect of genotype, *p* > 0.32 for total play^a15^ and pins^b15^), when play was low for all genotypes; levels of play at P17 were too low for statistical analyses.

### Experiment 2: social behaviors and ultrasonic vocalizations of juvenile Brattleboro rats

#### Social behaviors

**Figure 2.**
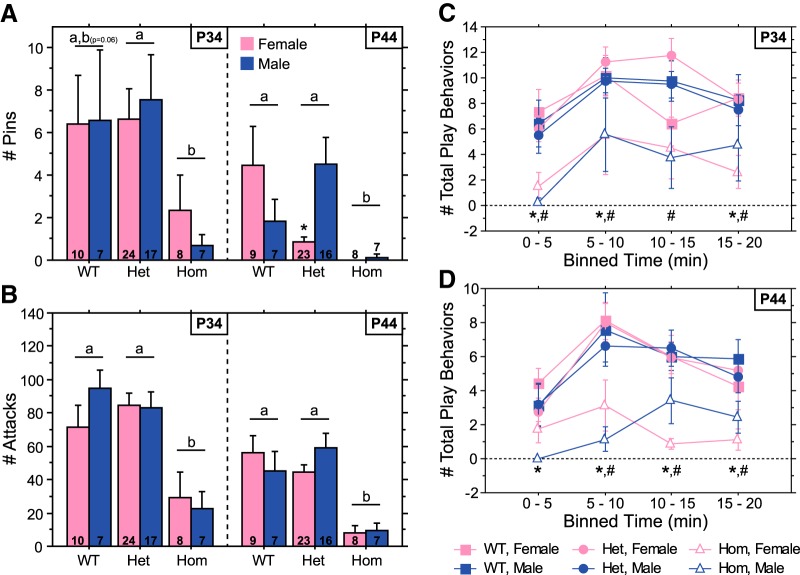
Social play is decreased in Hom Brattleboro juveniles. ***A***, ***B***, Number of pins (***A***) and play attacks (***B***) of male and female Hom, Het, and WT rats during a 20 min test at P34 or P44. ***C***, ***D***, The temporal profile of play is illustrated in ***C*** (for P34) and ***D*** (for P44) as the number of total play behaviors binned every 5 min. Sample sizes are indicated within each bar in ***A*** and ***B***. Data from each age were obtained from separate cohorts of animals. Genotypes with differing letters differ significantly from each other (*p* < 0.05, Fisher’s PLSD); where differences approach significance, the *p* value is included in parentheses next to the letter representing the appropriate comparison. In ***C*** and ***D***, significant differences between Hom and WT or Het rats within each bin are indicated by * and #, respectively (*p* < 0.005, Fisher’s PLSD). See Results for ANOVA details.

**Figure 3.**
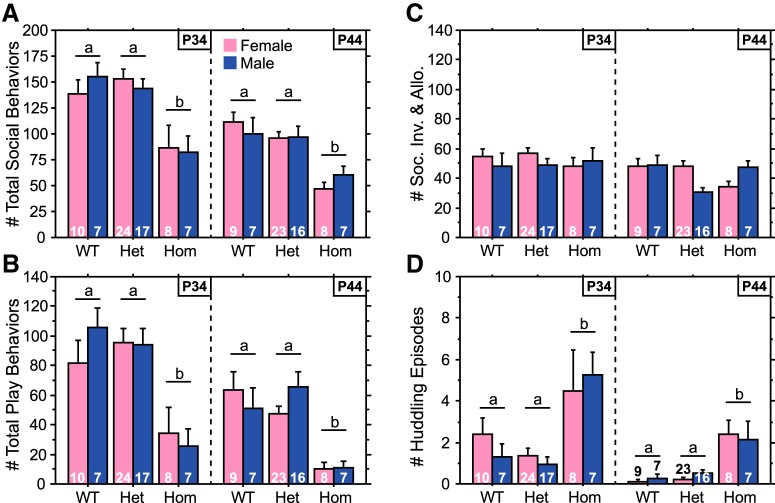
Social behavior is altered in Hom Brattleboro juveniles. ***A–D***, Number of total social behaviors (***A***), total play behaviors (***B***), social investigation & allogrooming behaviors (***C***), and huddling episodes (***D***) of Hom, Het, and WT rats during a 20 min test at P34 or P44. Sample sizes are indicated within each bar. Data from each age were obtained from separate cohorts of animals. Genotypes with differing letters differ significantly from each other (*p* < 0.05, Fisher’s PLSD). See Results for ANOVA details.

As for weanling-aged rats, Hom juveniles played less than WT and Het juveniles due to reductions in both pins and play attacks (Figs. 2, 3*B*; main effect of genotype, *p* < 0.0001 for total play^d2^, pins^e2^, and play attacks^f2^; Hom vs WT^d4,e4,f4^ or Het^d5,e5,f5^, *p* < 0.005 for all three behaviors). Analysis of the temporal profile of total play revealed that Hom juveniles played less than WT and Het rats across the entire 20 min test (Fig. 2*C*,*D*). Hom juveniles exhibited fewer total social behaviors than WT and Het juveniles (Fig. 3*A*; main effect of genotype, *p* < 0.0001^g2^; Hom vs WT^g4^ or Het^g5^, *p* < 0.0001). Reductions in play and social behaviors of Hom juveniles were evident at both P34 and P44, although the *post hoc* comparison between WT and Hom groups for pins at P34 fell short of significance when each age was analyzed separately (Figs. 2, 3). Social investigation & allogrooming did not differ between genotypes (Fig. 3*C*; main effect of genotype, *p* > 0.51^h2^), and huddling episodes were increased in Hom juveniles (Fig. 3*D*; main effect of genotype, *p* < 0.0001^i2^; Hom vs WT^i4^ or Het^i5^, *p* < 0.0001), indicating that not all social behaviors are affected in the same manner by the Brattleboro mutation.

The number of all social behaviors decreased between P34 and P44 (Figs. 2, 3; main effect of age, *p* < 0.01 for total play^d1^, pins^e1^, play attacks^f1^, total social behaviors^g1^, social investigation & allogrooming^h1^, and huddling^i1^). There were no significant sex differences in the number of social behaviors (main effect of sex, *p* > 0.46 for total play^d3^, pins^e3^, play attacks^f3^, total social behaviors^g3^, social investigation & allogrooming^h3^, and huddling^i3^), except that male Het juveniles exhibited more pins than female Het juveniles at 44 d of age (Fig. 2*B*; genotype × sex interaction at P44, *p* < 0.008^e6^; Het male vs Het female, *p* < 0.002^e7^).

#### Ultrasonic vocalizations

**Figure 4.**
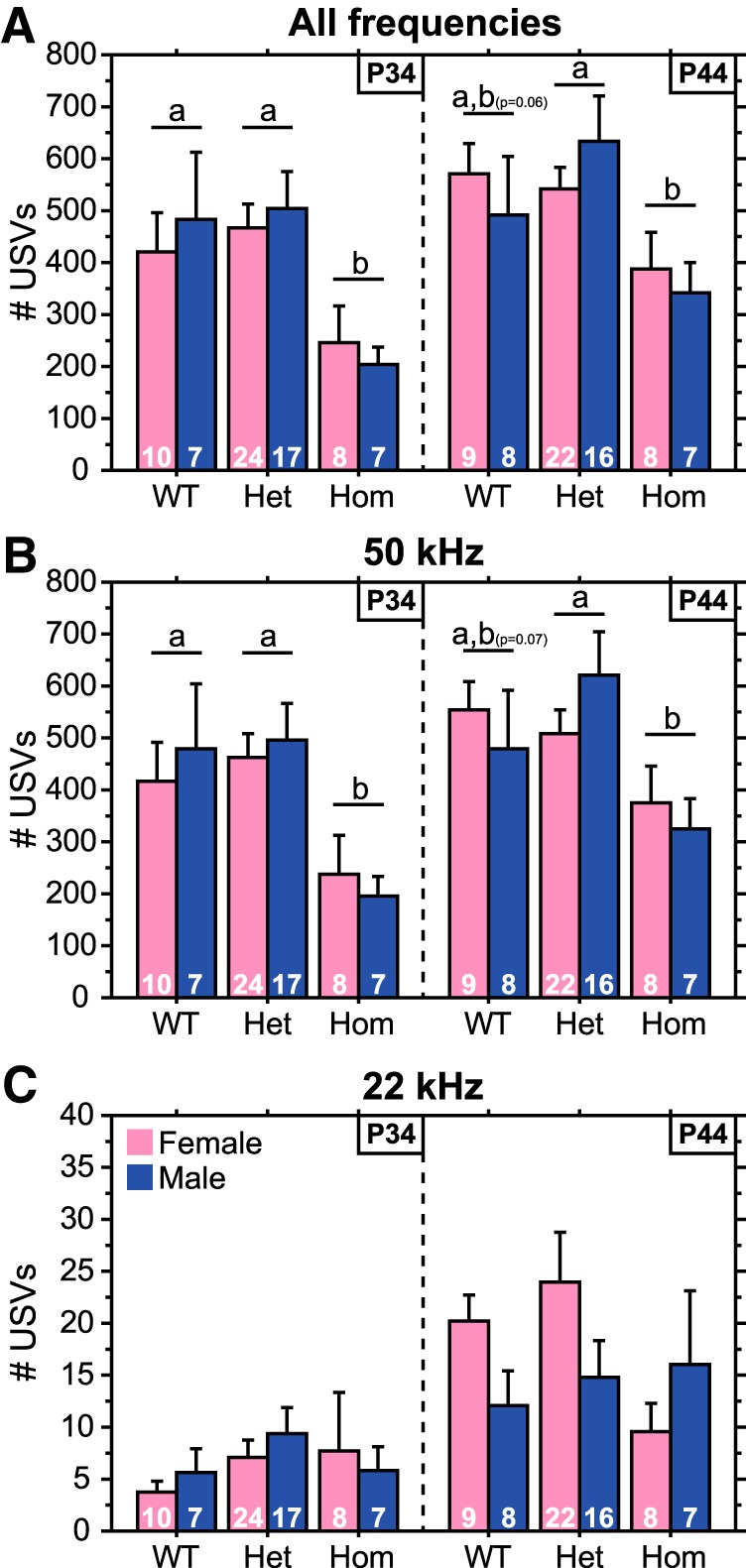
Hom Brattleboro juveniles emit fewer 50 kHz USVs. ***A–C***, Number of all (***A***), 50 kHz (***B***), and 22 kHz (***C***) USVs of male and female Hom, Het, and WT rats during the first 10 min of a 20 min test at P34 or P44. Sample sizes are indicated within each bar. Data from each age were obtained from separate cohorts of animals. Genotypes with differing letters differ significantly from each other (*p* < 0.05, Fisher’s PLSD); where differences approach significance, the *p* value is included in parentheses next to the letter representing the appropriate comparison. See Results for ANOVA details.

Similar to play and overall social behaviors, Hom rats emitted fewer USVs than WT and Het rats (Fig. 4; main effect of genotype, *p* < 0.0001^j1^; Hom vs WT^j2^ or Het^j3^, *p* < 0.003) due to a selective reduction in 50 kHz USVs (Fig. 4*B*,*C*; 50 kHz USVs: main effect of genotype, *p* < 0.0001^k2^; Hom vs WT^k3^ or Het^k4^, *p* < 0.002; 22 kHz USVs: main effect of genotype, *p* > 0.25^l2^). Decreased 50 kHz USVs of Hom rats were evident at both juvenile ages, although the *post hoc* comparisons between WT and Hom rats fell short of significance when P44 data were analyzed separately (Fig. 4*B*). Unlike social behaviors, the number of 50 and 22 kHz USVs increased across age (main effect of age, *p* < 0.04 for 50 kHz and 22 kHz USVs).

The 50 kHz USV category consists of calls with a broad range of frequencies (30–117 kHz in the present study) and spectral-temporal structures (e.g., constant frequency, frequency steps, frequency trills), and it is not known whether these calls are functionally equivalent. To determine which types of 50 kHz USVs were impacted by the Brattleboro mutation, we classified a subset (20%) of the USVs of each animal according to the call types proposed by Wright et al. (2010) and assessed the impact of the Brattleboro mutation on the quantity (number) and quality (duration and integrated frequency, which was defined as the mean of the call onset, peak amplitude, and call end frequency) of the vocalizations most frequently emitted during social behavior testing.

**Figure 5.**
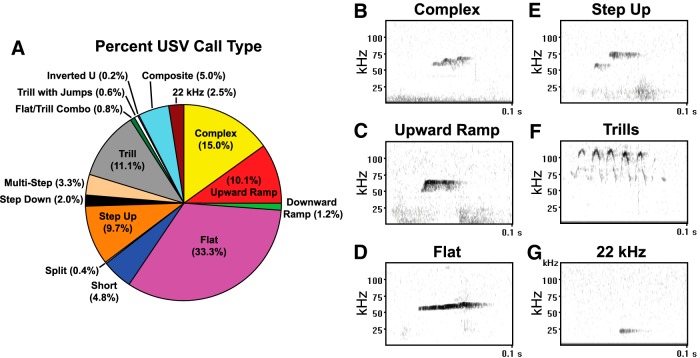
Ultrasonic vocalization call types emitted during social behavior testing. ***A***, Percentage of USV call types, as defined by Wright et al. (2010), emitted by male and female Hom, Het, and WT juveniles (P34 or P44) during the first 10 min of a 20 min test; data are combined across sex, genotype, and age. ***B–G***, Representative spectrograms of the most common 50 kHz calls [complex (***B***), upward-ramp (***C***), flat (***D***), step-up (***E***), and trill (***F***)] as well as the 22 kHz call (***G***).

Figure 5 illustrates the percentage of all classified calls, regardless of the genotype of the caller. Most USVs fell within the 50 kHz category, with type 4 calls (flat calls) being the most common (33.3%) followed by type 1 (complex calls, 15.0%), type 10 (trills, 11.1%), type 2 (upward-ramp calls, 10.1%), and type 7 calls (step-up calls, 9.7%). The percentage for each of the remaining call types was <5%, including 22 kHz USVs (type 15), which comprised 2.5% of calls.

#### Quantity of ultrasonic vocalization call types

**Figure 6.**
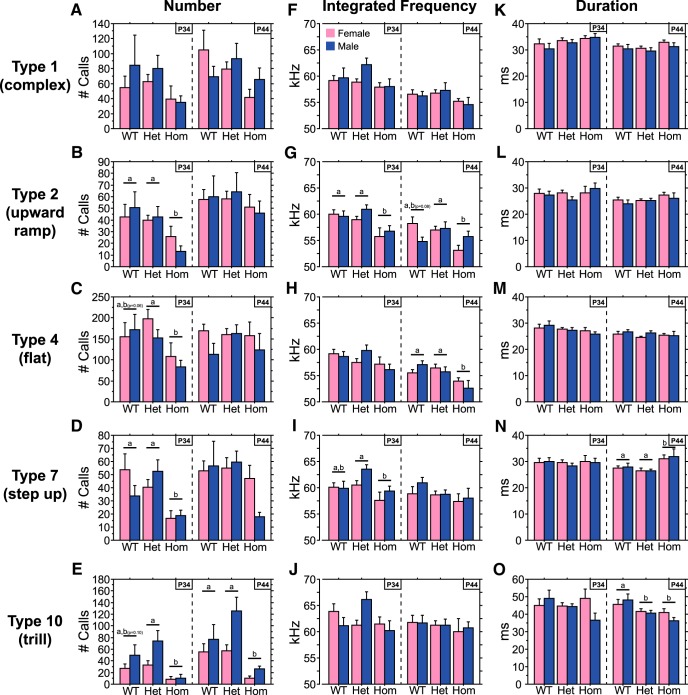
The quantity and quality of USV calls is altered in Hom Brattleboro rats. ***A–O***, number (***A–E***), integrated frequency (***F–J***), and duration (***K–O***) of type 1 (complex), type 2 (upward-ramp), type 4 (flat), type 7 (step-up), and type 10 (trill) USV calls of male and female Hom, Het, and WT rats during the first 10 min of a 20 min test at P34 or P44. Data from each age were obtained from separate cohorts of animals. Genotypes with differing letters differ significantly from each other (*p* < 0.05, Fisher’s PLSD); where differences approach significance, the *p* value is included in parentheses next to the letter representing the appropriate comparison. See Results for ANOVA details.

In general, juvenile Hom rats emitted fewer calls of each type than their WT and Het littermates, and for most this was due to a decreased call number at P34 (Fig. 6*A–E*). At P34, Hom rats emitted fewer upward-ramp, flat, and step-up calls (Fig. 6*B–D*; main effect of genotype, *p* < 0.03 for each call type^n2,o2,p2^; Hom vs WT, *p* < 0.02 for upward-ramp^n3^ and step-up^p3^ calls; Hom vs Het, *p* < 0.02 for each call type^n4,o4,p4^), although the comparison between WT and Hom flat calls fell short of significance (*p* = 0.06^°3^). At P44, there were no significant differences between genotypes for these calls (main effect of genotype, *p* > 0.05 for upward-ramp^n5^, flat^o5^, and step-up^p5^ calls). Trills were reduced in Hom juveniles at both P34 and P44 (Fig. 6*E*; main effect of genotype, *p* < 0.01 for both P34^q3^ and P44^q6^; Hom vs WT, *p* < 0.04 for P44^q7^; Hom vs Het, *p* < 0.005 for both P34^q5^ and P44^q8^), although the difference between Hom and WT rats was not significant at P34 (*p* = 0.10^q4^). When analyzed across both ages, Hom rats emitted fewer complex calls than WT and Het rats (main effect of genotype, *p* < 0.03^m2^; Hom vs WT ^m3^or Het^m4^, *p* < 0.04), but these comparisons were not significant when assessed separately for each age (Fig. 6*A*; main effect of genotype, *p* > 0.15 for P34^m5^ and P44^m6^).

Upward-ramp, step-up, and trill calls increased from P34 to P44 (Fig. 6*B*,*D*,*E*; main effect of age, *p* < 0.04 for each call type^n1,p1,q1^); complex and flat calls did not differ across age (Fig. 6*A*,*C*; main effect of age, *p* > 0.13 for both calls^m1,o1^). Only trills differed between the sexes, with males emitting more than females (Fig. 6*E*; main effect of sex, *p* < 0.005^q2^).

#### Quality of ultrasonic vocalization call types

For most USV types, Hom rats emitted calls with a lower integrated frequency, but duration was only altered for step-up calls and trills. Upward-ramp, flat, and step-up calls of Hom rats had a lower integrated frequency than those of WT and Het rats (main effect of genotype, *p* < 0.02 for each call type^s1,t1,u1^; Hom vs WT^s2,t2,u2^ or Het^s3,t3,u3^, *p* < 0.05 for each call type). The age at which these effects were significant depended on the call type (Fig. 6*G–I*). Complex calls of Hom rats also had a lower integrated frequency than those of Het rats (main effect of genotype, *p* < 0.02^r1^; Hom vs Het, *p* < 0.006^r3^), but did not differ significantly from WT rats (*p* = 0.10^r2^); Hom and Het differences in complex calls were not significant when those from P34 and P44 were analyzed separately (Fig. 6*F*). Trills were the only call type analyzed for which integrated frequency was unaffected by genotype (Fig. 6*J*; main effect of genotype, *p* > 0.24^v1^).

The duration of USVs also differed by genotype, but only for step-up calls and trills, and only at P44 (Fig. 6*K–O*). Hom rats emitted longer duration step-up calls than WT and Het rats at P44 (Fig. 6*N*; main effect of genotype, *p* < 0.003 for P44^w1^; Hom vs WT^w2^ or Het^w3^, *p* < 0.02). For trills, both Hom and Het rats emitted shorter calls than WT rats at P44, and Hom and Het rats did not differ from each other (Fig. 6*O*; main effect of genotype, *p* < 0.01^x1^; Hom^x2^ or Het^x3^ vs WT, *p* < 0.02; Het vs Hom, *p* > 0.26^x4^).

The quality of USV call types also changed with age. The integrated frequency decreased from P34 to P44 for each call type except trills (Fig. 6*F–J*; main effect of age, *p* < 0.05 for complex^r4^, upward-ramp^s4^, flat^t4^, and step-up^u4^ calls; main effect of age, *p* > 0.18 for trills^v2^). This reduction in integrated frequency was evident in all genotypes; no interaction between age and genotype was found (age × genotype, *p* > 0.05 for complex^r5^, upward-ramp^s5^, flat^t5^, and step-up^u5^ calls). In addition, the duration of complex, upward-ramp, and flat calls also decreased from P34 to P44 (Fig. 6*K–M*; main effect of age, *p* < 0.03 for each call type^y1,z1,aa1^); the duration of step-up calls and trills did not vary with age (Fig. 6*N*,*O*; main effect of age, *p* > 0.10 for step-up calls^w4^ and trills^x5^).

## Discussion

The present study suggests that the *Avp* gene plays an important role in social development. The Brattleboro mutation, which disrupts the production of AVP, impacted both social behaviors and ultrasonic communication of juvenile rats. Hom rats played less and emitted fewer 50 kHz USVs than their WT and Het littermates. In addition, the spectrotemporal characteristics of USVs emitted by Hom rats differed from those of WT and Het rats. Social deficits, however, were behavior and USV specific. Huddling episodes were increased in Hom rats, and social investigation & allogrooming, and 22 kHz USVs did not differ across genotypes. Hence, Hom Brattleboro rats are not simply asocial, rather their social behaviors are “atypical” compared with those of WT and Het rats.

Deficits in the social play of Hom rats were evident throughout the developmental profile of play [onset (P21 and P23), peak (P34), and decline (P44)], suggesting that AVP is important for the overall level of play rather than its developmental timing. This is similar to the persistent developmental deficits reported for body and brain weights of Hom Brattleboro rats (for review, see Boer, 1985), but differs from other measures (e.g., eye opening, ear opening, incisor eruption), which occur earlier in Hom Brattleboro rats (Boer et al., 1980; Zelena et al., 2009). Notably, the greatest deficits in the neural development of Hom Brattleboro rats occur in the cerebellum (Boer et al., 1982), a brain region whose development correlates with the ontogeny of play across several species (Byers and Walker, 1995). It has been proposed that play behavior contributes to cerebellar development (Byers and Walker, 1995). Following this logic, it is possible that the decreased play of Hom rats contributes to developmental deficits in their cerebellar size and morphology. The reverse, however, is also possible.

Adult Het rats exhibit partial reductions in AVP neural mRNA expression and pituitary peptide content (Dorsa and Bottemiller, 1982), and sometimes exhibit behavioral differences from WT rats (Brot et al., 1992). Nonetheless, the social behaviors of Het rats in the present study did not differ statistically from those of WT rats except for a transient reduction in social play during the developmental onset of play (at P21). These data raise the possibility of a gene dosage effect during the developmental onset of play. Perhaps the onset of play requires higher levels of AVP than its maintenance or Het rats have insufficient AVP at this age to stimulate play.

The Brattleboro mutation also affects USVs. Infant Hom Brattleboro pups emit fewer maternal separation-induced 40 kHz USVs (Lin et al., 2013; Varga et al., 2015). Here, we demonstrate that USV deficits of Hom Brattleboro rats persist into the juvenile stage and include prosocial vocalizations. Juvenile Hom rats emitted fewer USVs during the social interaction test due to a selective reduction in 50 kHz calls. Fifty kilohertz USVs reflect a positive affective state and are considered a form of prosocial communication (for review, see Brudzynski, 2013; Wöhr and Schwarting, 2013). Fifty kilohertz calls are emitted during appetitive interactions and in anticipation of reward stimuli, such as mating, play, addictive drugs, and “tickling” (Barfield et al., 1979; Knutson et al., 1998, 1999; Panksepp and Burgdorf, 2000; Burgdorf et al., 2008). Furthermore, 50 kHz calls elicit approach behavior (Wöhr and Schwarting, 2007) and “self administration” for their playback (Burgdorf et al., 2008). Hence, decreased 50 kHz USVs in Hom Brattleboro rats may indicate decreased prosocial motivation for, or reward value of, social interactions in AVP-deficient animals.

In contrast, 22 kHz calls are emitted in response to aversive stimuli (e.g., electric shocks, predators, drug withdrawal, and aggressive interactions; Sales, 1972; Tonoue et al., 1986; Cuomo et al., 1988; Blanchard et al., 1991; Vivian and Miczek, 1991; Barros and Miczek, 1996; Covington and Miczek, 2003), are thought to reflect a negative affective state akin to anxiety or distress (for review, see Brudzynski, 2013; Wöhr and Schwarting, 2013), and are not affected by the Brattleboro mutation (present findings). Hence, AVP deficiency does not affect all forms of vocal communication, with distress-like calls being particularly independent of AVP status. This conclusion should be tempered by the low levels of 22 kHz calls for all genotypes in the present experiment, which is consistent with the findings of previous studies measuring USVs during prosocial playful interactions (Burgdorf et al., 2006). In addition, while the 22 kHz USVs in the present study were within the frequency range of distress-like USVs, their duration was much shorter than that typically reported: ∼24 ms in the current study (Fig. 5*G*) versus 300–1200 ms in studies investigating USVs in response to aversive stimuli (Tonoue et al., 1986; Brudzynski and Ociepa, 1992). Brudzynski et al. (1993) reported two distinct populations of 22 kHz USVs in response to experimenter handling: short calls of 20–300 ms and long calls of 300 to >2000 ms, with most long calls falling between 500 and 600 ms. The functional significance of these short 22 kHz calls is not known. Therefore, it is possible that the 22 kHz USVs in the present study were not true anxiety or distress-like calls. Future studies are needed to determine whether 22 kHz USVs are altered in Hom Brattleboro rats tested under aversive conditions.

We further analyzed the USVs according to subcategories suggested by Wright et al. (2010) to determine whether the Brattleboro mutation differentially impacted different types of calls (i.e. altered their vocal repertoire). In general, Hom Brattleboro rats emitted fewer of each USV call type analyzed. Reductions were evident in each of the five following most common call types: flat calls, complex calls, trills, upward-ramp calls, and step-up calls (all 50 kHz calls). Deficits were most robust for trills, which were present at both P34 and P44, and least robust for complex calls, which were not significant when each age was analyzed separately. In addition to the quantity of USVs, the Brattleboro mutation impacted spectrotemporal characteristics of USVs. Flat, upward-ramp, and step-up calls of Hom rats had lower integrated frequencies. In addition, step-up calls were longer and trills were shorter in Hom rats. While it is not clear why AVP deficiency impacts the spectrotemporal quality of USVs in a call-specific manner, it is clear that several USV call types of Hom rats sound different than those of WT and Het rats. It is interesting to speculate that the reduced number and integrated frequency of 50 kHz USVs of Hom rats may contribute to their “atypical” social behaviors. Call frequency is an important feature for USV call structure. Frequency is the dominant feature mice use to discriminate between tone categories (Radziwon and Dent, 2014). In addition, rats will approach a speaker playing 50 kHz calls and tones (Wöhr and Schwarting, 2007). Hence, the decreased integrated frequency of flat, upward-ramp, and step-up calls of Hom rats might impact call meaning or appetitive quality. Therefore, the reduced number and frequency of 50 kHz calls might lead to less play by reducing the amount of prosocial stimulation during social interactions. This rationale, however, cannot explain the increased huddling seen in Hom rats. Perhaps the prosocial nature of 50 kHz USVs depends upon the type of social behavior. For example, because 50 kHz USVs stimulate locomotor behavior (Wöhr and Schwarting, 2007), it is possible that they stimulate “active” social behaviors such as play, but inhibit “passive” social behaviors such as huddling. It should be noted that a direct relationship between the number of USVs and play events has not been established, and it is also possible that play triggers USVs. Our data suggest that a simple relationship between play and USVs as a whole is unlikely; in Experiment 2, for example, the number of USVs increased with age, whereas the number of play events (and social interactions) decreased with age.

Despite significant interest in adolescent social development, most studies on the development of USVs have focused on infant maternal separation-induced 40 kHz calls. A few reports have found that adult rodents emit more USVs than adolescents during same-sex or opposite-sex interactions (Cherry, 1987; Willey et al., 2009; Willey and Spear, 2012; Kabitzke et al., 2015). Similarly, we found that the number of both 50 and 22 kHz USVs emitted during same-sex juvenile social interactions increase across a short 10 d interval from P34 to P44, which approximates early/mid-adolescence in rats (Vetter-O’Hagen and Spear, 2012). We further found that the developmental increases in 50 kHz USVs were specific to call type, occurring in upward-ramp, step-up, and trill calls, but not complex or flat calls. In addition, the spectrotemporal characteristics of several 50 kHz call types changed across these ages: integrated frequency decreased for complex, upward-ramp, flat, and step-up calls; and duration decreased for complex, upward-ramp, and flat calls. These findings raise the possibility that spectrotemporal characteristics of some rat USVs convey age-related information of the caller and thereby influence age-dependent social interactions. For example, perhaps the spectrotemporal characteristics of prepubertal calls elicit less aggression from same-sex adults, whereas those of postpubertal calls may better stimulate sex behaviors in the opposite sex.

Males emit more USVs than females, both as infants in response to maternal separation (Bowers et al., 2013), and as juveniles immediately preceding and following play bouts (Himmler et al., 2014). In the present experiment, we found that the sex difference in the number of USVs during juvenile play is restricted to trills, with males again emitting more than females. Although Himmler et al. (2014) did not report whether sex differences were present in all or some USV call types, trills comprised 77% of calls in their analysis. We did not detect any sex differences in the integrated frequency or duration of any call type, including trills, suggesting that juvenile sex differences in USVs are limited to quantity rather than spectrotemporal quality. With the exception of a single comparison in 44-d-old Het rats (Fig. 4*A*), we did not detect sex differences in other social behaviors, including play. Although males are often reported to engage in more rough-and-tumble play than females (Meaney and Stewart, 1981b; Pellis, 2002; Olesen et al., 2005), this sex difference is dependent upon the testing conditions and behaviors measured (Thor and Holloway, 1984; Argue and McCarthy, 2015). The absence of sex differences in play in the present experiments is not surprising as studies testing same-sex pairs of rats after a period of isolation, as we did in the present experiments, generally do not detect sex differences in juvenile social play (Panksepp and Beatty, 1980; Panksepp, 1981; Veenema et al., 2013; Paul et al., 2014).

Currently, we cannot determine whether the altered social behavior and USVs of Brattleboro rats are due to the absence of central or peripheral actions of AVP. Hom Brattleboro rats develop diabetes insipidus due to the absence of AVP-mediated water reabsorption at the level of the kidney (Valtin and Schroeder, 1964), and symptoms are evident before play onset: increased plasma osmolarity is present at 10-14 d of age, and polydipsia develops between 15 and 16 d of age (Dlouhá et al., 1982; Zelena et al., 2009). Nonetheless, acute intracerebroventricular injections of a vasopressin 1a receptor (V1aR) antagonist decrease maternal separation-induced 40 kHz USVs of infant rat pups (Winslow and Insel, 1993; Bleickardt et al., 2009), as well as 50 kHz USVs and play behavior of male juvenile rats (Veenema et al., 2013; Lukas and Wöhr, 2015). These findings argue that the altered play and USVs of Brattleboro rats is due to a direct disruption of the actions of AVP in the brain rather than a disruption of the peripheral actions of AVP or indirect compensatory changes resulting from the absence of AVP during development. Decreased anxiety-like, depressive-like, and maternal behaviors of Brattleboro rats persist after the restoration of the peripheral actions of AVP, indicating that several behavioral abnormalities of Brattleboro rats are due to the loss of the central actions of AVP (Fodor et al., 2012; Balázsfi et al., 2015).

We do not yet know which AVP system is responsible for the deficits seen in the Brattleboro rats. The limited available data focus on juvenile social play and do not provide a comprehensive understanding of the regulation by AVP of social development. AVP cells in the bed nucleus of the stria terminalis (BNST) appear to play an inhibitory, rather than stimulatory, role in juvenile social play, at least in males. During the developmental emergence in weanling-aged rats (P18–P23), BNST AVP mRNA expression of males correlates negatively with play behavior, whereas in females, BNST AVP mRNA expression is not detectable (Paul et al., 2014). Furthermore, V1aR antagonist injections into the septum, a projection area of BNST AVP cells, increases the play behavior of juvenile males, but not females (Veenema et al., 2013). In the same study, intracerebroventricular V1aR antagonist injections decreased the play behavior of male rats but increased the play behavior of female rats. AVP mRNA expression in the paraventricular nucleus of the hypothalamus (PVN) of male, but not female, rats correlates positively with their play behavior during the developmental emergence of play, raising the possibility that the PVN is the site of the stimulatory actions of AVP, at least in males (Paul et al., 2014). At present, however, it is difficult to incorporate these sex-specific findings with the present results in which “atypical” social behaviors of Hom rats were seen in both sexes (including play deficits). Complicating matters further, play behavior and the effects of pharmacological manipulations of AVP on play can depend upon the context in which the animals are tested (Bredewold et al., 2014). AVP in the PVN regulates the stress axis and autonomic function, both of which could influence social behavior in a context-specific manner through their actions on stress or arousal. Adult Brattleboro rats are less reactive to various stressors (Balázsfi et al., 2015). If true for juveniles, this decreased stress reactivity could have contributed to the present findings where play was tested in a novel home cage after a period of isolation. Although rats play less after restraint stress (Klein et al., 2010), isolation increases play in rats (Panksepp and Beatty, 1980). Hence, it is not clear whether decreased stress reactivity would lead to higher or lower levels of play. The low levels of 22 kHz USVs (and the absence of long 22 kHz USVs) across all genotypes suggest that rats were not distressed or anxious in the novel cage during testing. Genotype differences in novel cage exploration at the beginning of the test are unlikely to account for the decreased play as Hom Brattleboro rats exhibited lower levels of play across the entire 20 min test (Fig. 2*C*,*D*). More studies are needed to dissect out which AVP systems contribute to social development and how.

Early life experiences can significantly impact behavioral development (Kundakovic and Champagne, 2015), and juvenile social play is particularly sensitive to both prenatal and early postnatal environments (Veenema and Neumann, 2009; Kirsten et al., 2010; Taylor et al., 2012; Karkow and Lucion, 2013), including natural variations in maternal care (Parent and Meaney, 2008). Given that the Brattleboro mutation impacts maternal behaviors (Fodor et al., 2012), care must be taken when designing and interpreting results using this model. Indeed, Hom Brattleboro dams influence several behavioral and physiological characteristics of their offspring (e.g., body weight, brain weight, startle response, and stress reactivity; Snijdewint et al., 1988; Zelena et al., 2003, 2009; Feifel and Priebe, 2007). To minimize the potential impact of the early-life environment in the present experiment, we tested Hom, Het, and WT littermates born to Het dams, thereby removing potential prenatal and postnatal confounds of maternal genotype. Nonetheless, we cannot rule out possible confounds of differential maternal or sibling treatment toward Hom Brattleboro pups.

While it is now clear that AVP influences juvenile social behaviors, we know very little about how AVP acts to regulate social development. This represents a critical gap in our knowledge as altered AVP function has been implicated in neurodevelopmental disorders such as autism spectrum disorders and schizophrenia [Heinrichs et al., 2009; Rubin et al., 2014 (and references therein)]. Notably, Hom Brattleboro rats exhibit behavioral abnormalities associated with schizophrenia and autism spectrum disorders, including decreased social interactions, social cognitive deficits, and attenuated prepulse inhibition, which is consistent with the hypothesis that AVP signaling is disrupted in these disorders (Engelmann and Landgraf, 1994; Feifel and Priebe, 2007; Feifel et al., 2009; Lin et al., 2013). The present findings add social play and vocal communication to this list. Our findings also provide insight into the role of AVP in social development. By measuring multiple social behaviors in the same experiment, we were able to demonstrate that long-term disruption of AVP production does not simply decrease overall levels of social behavior, but rather alters the type of social behavior the animal expresses, leading to an atypical rather than asocial phenotype. Future studies are needed to uncover the neural mechanisms through which AVP influences both normal and disordered social development.

## Synthesis

The decision was a result of the Reviewing Editor Jeffrey Blaustein and the peer reviewers coming together and discussing their recommendations until a consensus was reached. A fact-based synthesis statement explaining their decision and outlining what is needed to prepare a revision is listed below. The following reviewers agreed to reveal their identity: Yvon Delville, J. Curtis.

In general, the reviewers believe that the manuscript has merit. However, there are a number of important comments (see below). However after consultation, the reviewers and editor agree with the following important points:

1. The authors must either perform a more complete analysis of USVs as they relate to social play and other events or provide a compelling argument as to why this is not necessary. The reviewers suggest that the numbers of USVs should be adjusted to social play time or events.

2. Considering the shortcomings of using the Brattleboro rat to draw conclusions about the role of VP, the authors should be more cautious in attributing the effects to VP directly. Terms like "suggest" would be more appropriate.

3. A caveat regarding the possibility that there is a role of stress in behavioral phenotype is essential for reasons delineated in the reviews. The authors need to provide an explanation re: why the reader should not be concerned about the confound of testing in a new environment.

Reviews:

Reviewer 1

Major Issues:

1. The authors seem to like using the word "demonstrate" in their Abstract, Introduction and Discussion sections. I strongly recommend using more cautious words for this particular study. The data are consistent with a role of vasopressin in the control of these behaviors, but they do not demonstrate a hypothesis.

2. The reason these data do not demonstrate the role of vasopressin in these behaviors has to do with the type of study carried out by the authors. In essence, this study is similar to gene knockout experiments. Yes, a gene is not expressed, but the behavioral outcome does not necessarily prove a behavioral role for the missing peptide. Vasopressin is only one of several neurotransmitters or neuromodulators mediating social play and ultrasonic vocalizations. For instance, vasopressin is well known to interact with serotonin which is also associated with social behavior. The absence of vasopressin throughout development may result in over-expression of serotonin, which could be the direct cause explaining the behavior of Brattleboro rats. Over time, compensatory systems could also take over for vasopressin. I think the authors are lucky to be working within a developmental time period when serotonin systems are not fully mature. While the authors did acknowledge the possibility of compensatory systems either within or outside of the brain on p. 20, this sections could be developed further or at the very least the authors need to acknowledge that their data do not demonstrate a role for vasopressin.

3. As noted by the authors social play and the role of vasopressin in this behavior is context-dependent. I am glad the authors addressed behavior before and after weaning. However, I am not entirely satisfied with the behavioral procedures. Before weaning, animals were taken out of their litters and placed in a new environment and tested with a strange (unknown) individual. Obviously, if the animals respond to this type of stress differently, their social behavior would also be different. Rats are social animals. Being isolated is stressful to them, particularly early in development. The authors need to address possible differential responses to stress as a possible confounding variable in their experimental design. Perhaps observations of the entire litters would be useful, as long as it is possible to track specific individuals inside the litters. (JDB: Possibility of stress interaction should be discussed.)

4. The procedure used to test animals after weaning is not entirely trouble-free either. The issue of isolation stress is also relevant here. Typically a period of isolation enhances play fighting in rats, and that could be different in Brattleboro rats. Maybe the behavioral differences are not specifically related to social play itself. This other confounding variable needs to be addressed as well. Personally I would have preferred observations between cagemates inside their home cages, or in response to an intruder. (JDB: Possibility of stress interaction should be discussed.)

5. It would be useful to provide some information about the distribution of the genotypes inside the litters. Could variability in the datasets originate from variability in the genotypes? (JDB: This will not require a full analysis, just mention of the distribution and speculation re: variability in genotype.)

6. I am very pleased to note the complex analysis of ultrasonic recordings. It would have been nice to match it with an equally complex analysis of social play observations. Social interactions in juvenile or infant rats are not limited to huddling, attacks, pins, and allogrooming. I would recommend future studies to include defensive components of the behavior as described by Sergio Pellis.

Minor Issues:

1. There is no need to explain the outcome of the study at the end of the Introduction section. This is anticlimatic for the reader. A hypothesis or predictions would be sufficient.

2. The beginning of the Results section (line 186) mentions a consistency with other reports. Where are the references?

Perhaps a publication by Sergio Pellis? These are missing from the reference list.

3. I am not convinced the tables of ANOVAs are really necessary. They are just making me worried that some analyses will come up significant by chance.

4. line 391. from not form.

5. I am not sure I see any relevance for autism in these studies. As noted by the authors, the Brattleboro rats were not asocial. I would recommend the authors be more careful when relating to mental disorders in humans.

Reviewer 2

Minor details:

In figure 3c, the y-axis title is "#Soc. Inv./Allo. Behaviors", which at first glance suggests a ratio of the two measures. It might be clearer to use “and” or “&”.

“Emit” is misspelled in the caption for figure 4.

The Byers and Walker ref is missing page numbers

In the Feifel et al. ref, “brattelboro” should be capitalized unless it was lower case in the original

In general, go through the references to ensure consistency of capitalization, abbreviation of journal names, etc.

I am going to assume that the figures will be published in color. If not, the bars should be rendered more distinguishable. (JDB: Because readers often print in black and white, enhancing the contrast when printed black and white is desirable.)

Genotype is misspelled on lines a8 and b8 of table 1

Since the data were collected from different groups of animals at each time point, it would be more appropriate to describe the groups as e.g., P34 “or” P44, rather than as P34 “and” P44. The latter implies the same animals were tested at both time points

Other comments

The authors cite Himmler's report of sex differences in USVs before or after play bouts, suggesting that the timing of vocalizations may contain information. I don't know if the data collection methods would allow for assessment of the timing of USVs relative to the onset (or termination) of the various social interactions, but doing so may provide further insights. Similarly, it might be useful to see whether specific types of calls consistently accompany particular play behaviors. I will leave it to the authors/ editor to decide whether this is worth pursuing.

It also might be interesting to test whether the AVP-deficient rats differ in their latency to exhibit particular behaviors, especially when the frequency of a behavior decreases. Is it possible that the motivation to initiate playing differs, but that once initiated, play behavior does not differ between genotypes? As above, I will leave it to the authors/editor to decide whether this is worth pursuing. (JDB: This is something for the authors to consider, but not required by Editor.)

Although the authors mention differences Het and WT individuals in a few cases, relatively little attention is paid to this type of result. Is it possible that a “dose/response” effect may come into play. For example, in figure 2, it appears that the Het responses for both Pins and Attacks at day 44 may be “dose-dependent” in males but not in females. In figure 1, a similar pattern is seen, but for both sexes, and with the Het group significantly different from the Hom and WT groups for pins and nearly so for total play at 21 days. Once again, I will leave it to the authors/editor to decide whether this is worth pursuing. (JDB: This is something for the authors to consider, but not required by Editor.)
